# Supplementary material for: BMN 250, a fusion of lysosomal alpha-N-acetylglucosaminidase with IGF2, exhibits different patterns of cellular uptake into critical cell types of Sanfilippo syndrome B disease pathogenesis
Source: PLoS One. 2019 Jan 18;14(1):e0207836. doi: 10.1371/journal.pone.0207836 (PMC6338363; doi:10.1371/journal.pone.0207836)
Supplement: S1 File — (PPTX) [file pone.0207836.s001.pptx]

## Slide 1
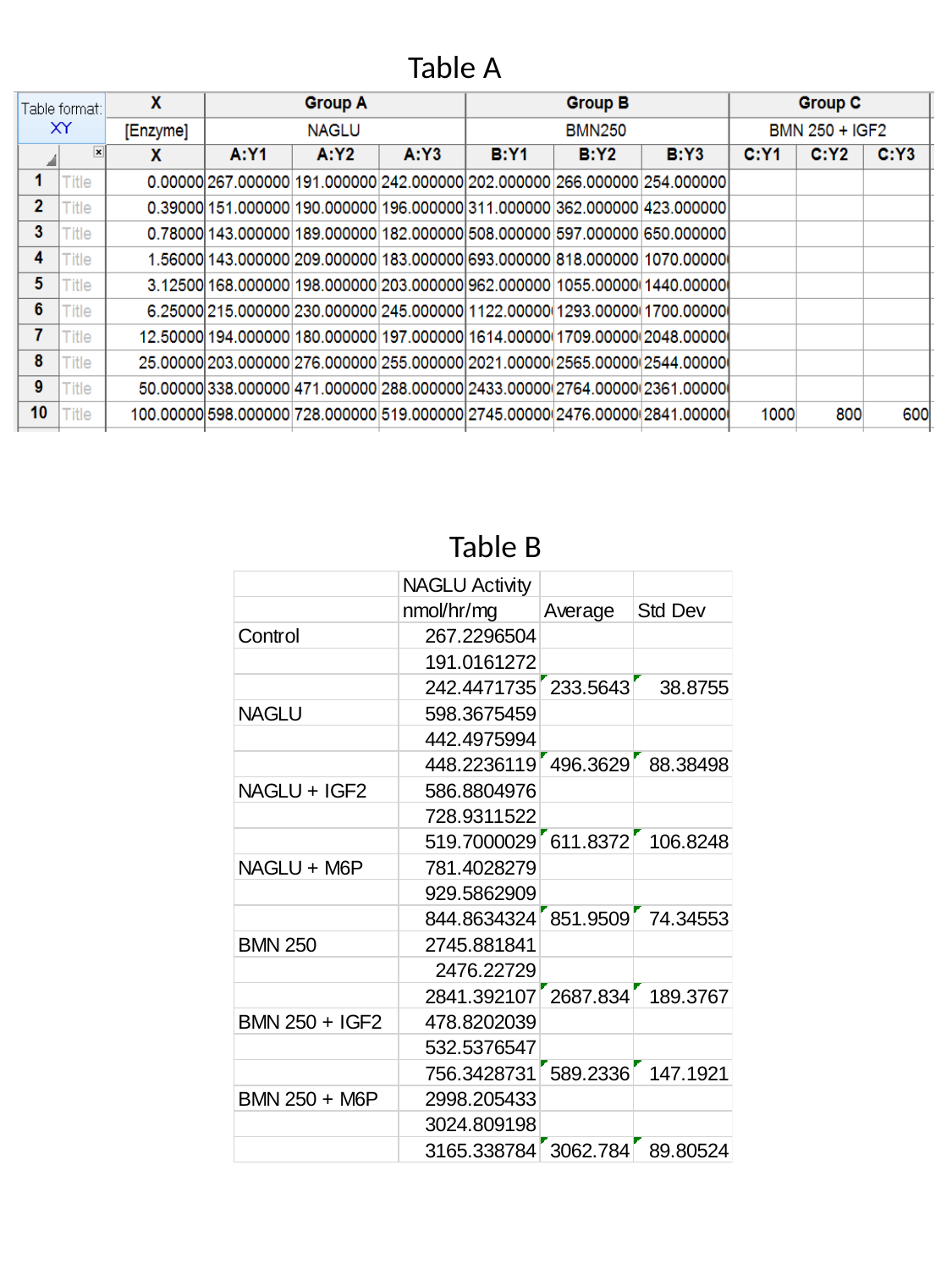

Table A
Table B

## Slide 2
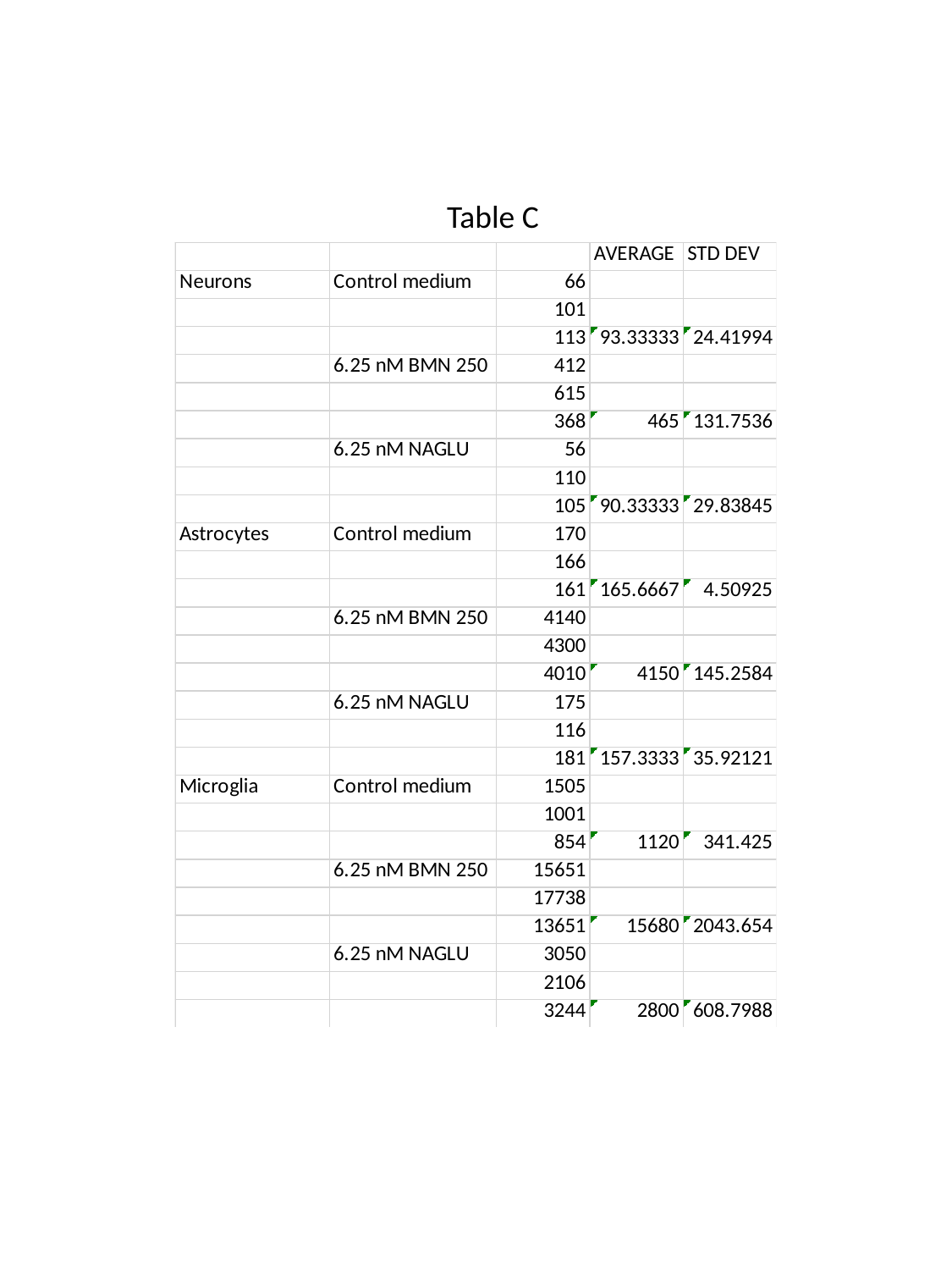

Table C

## Slide 3
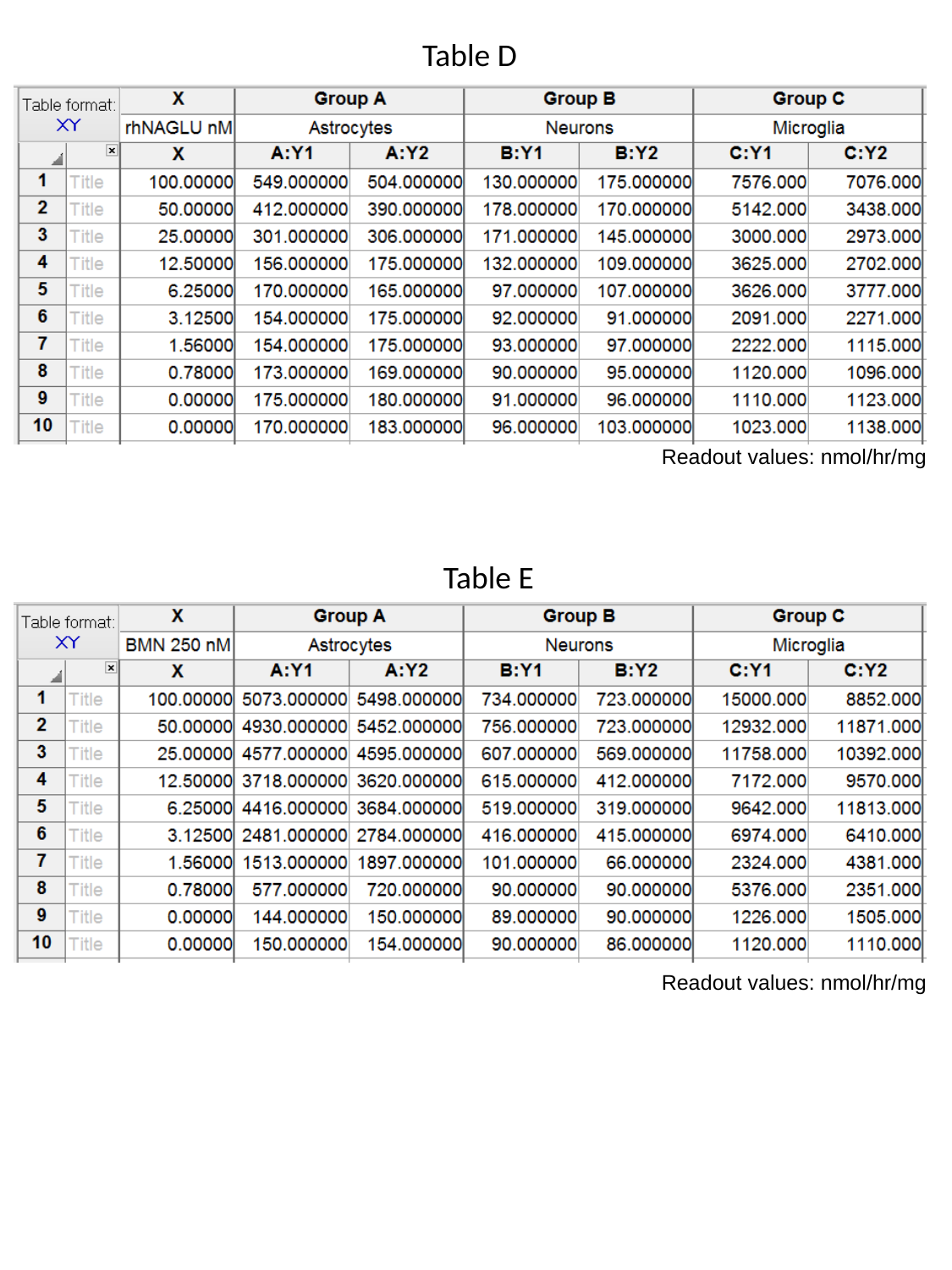

Table D
Readout values: nmol/hr/mg
Table E
Readout values: nmol/hr/mg

## Slide 4
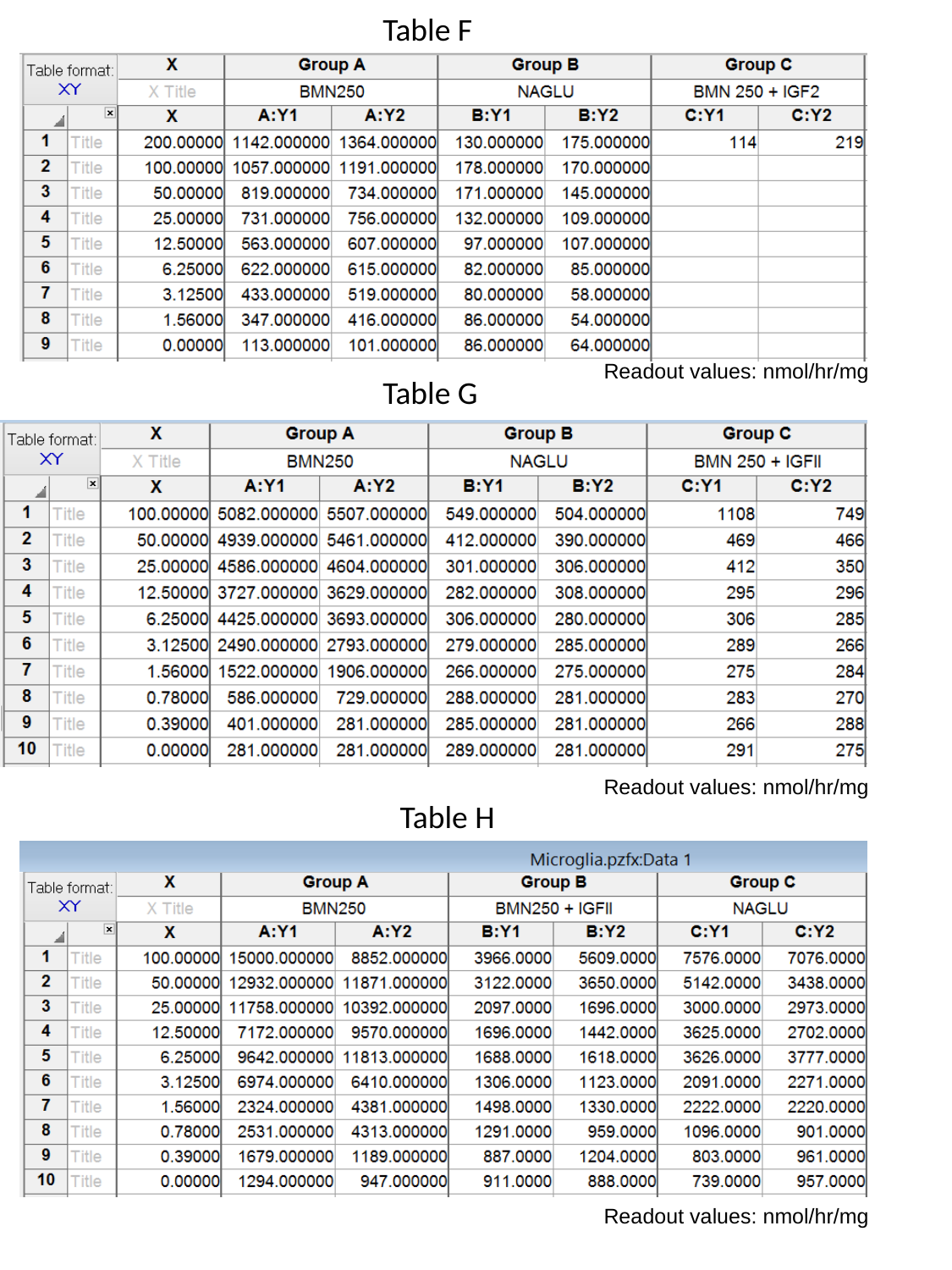

Table F
Readout values: nmol/hr/mg
Table G
Readout values: nmol/hr/mg
Table H
Readout values: nmol/hr/mg

## Slide 5
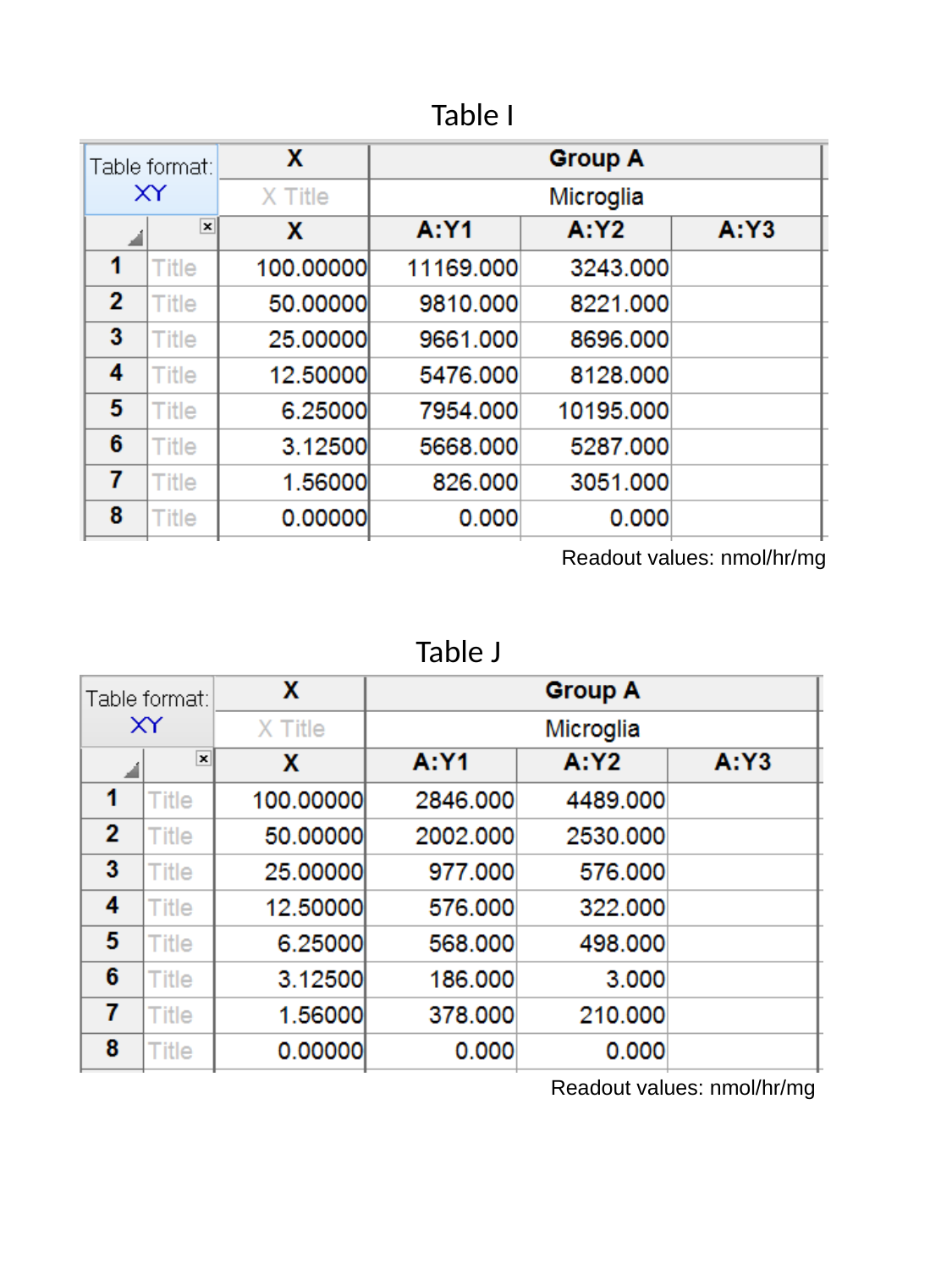

Table I
Readout values: nmol/hr/mg
Table J
Readout values: nmol/hr/mg

## Slide 6
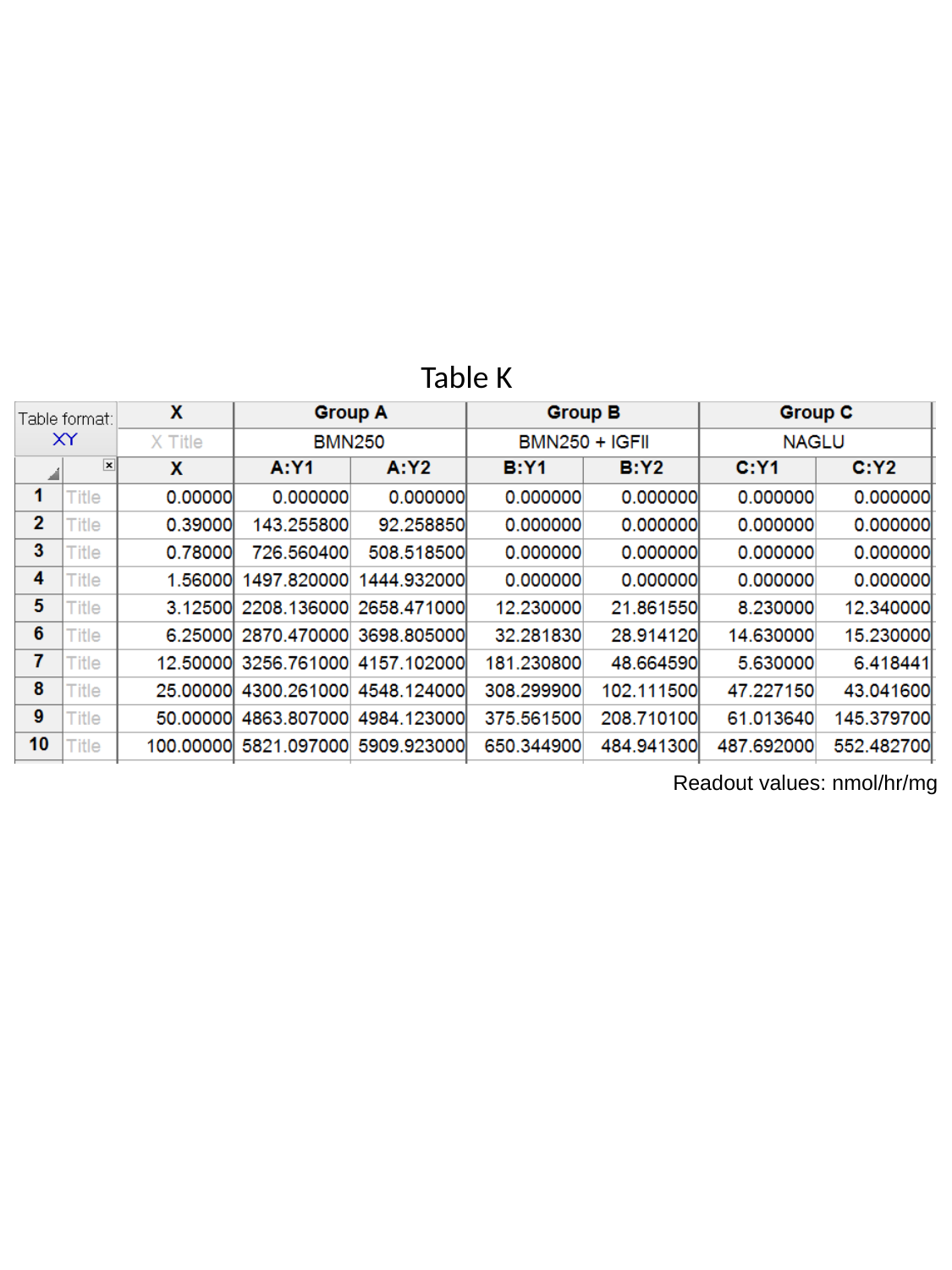

Table K
Readout values: nmol/hr/mg

## Slide 7
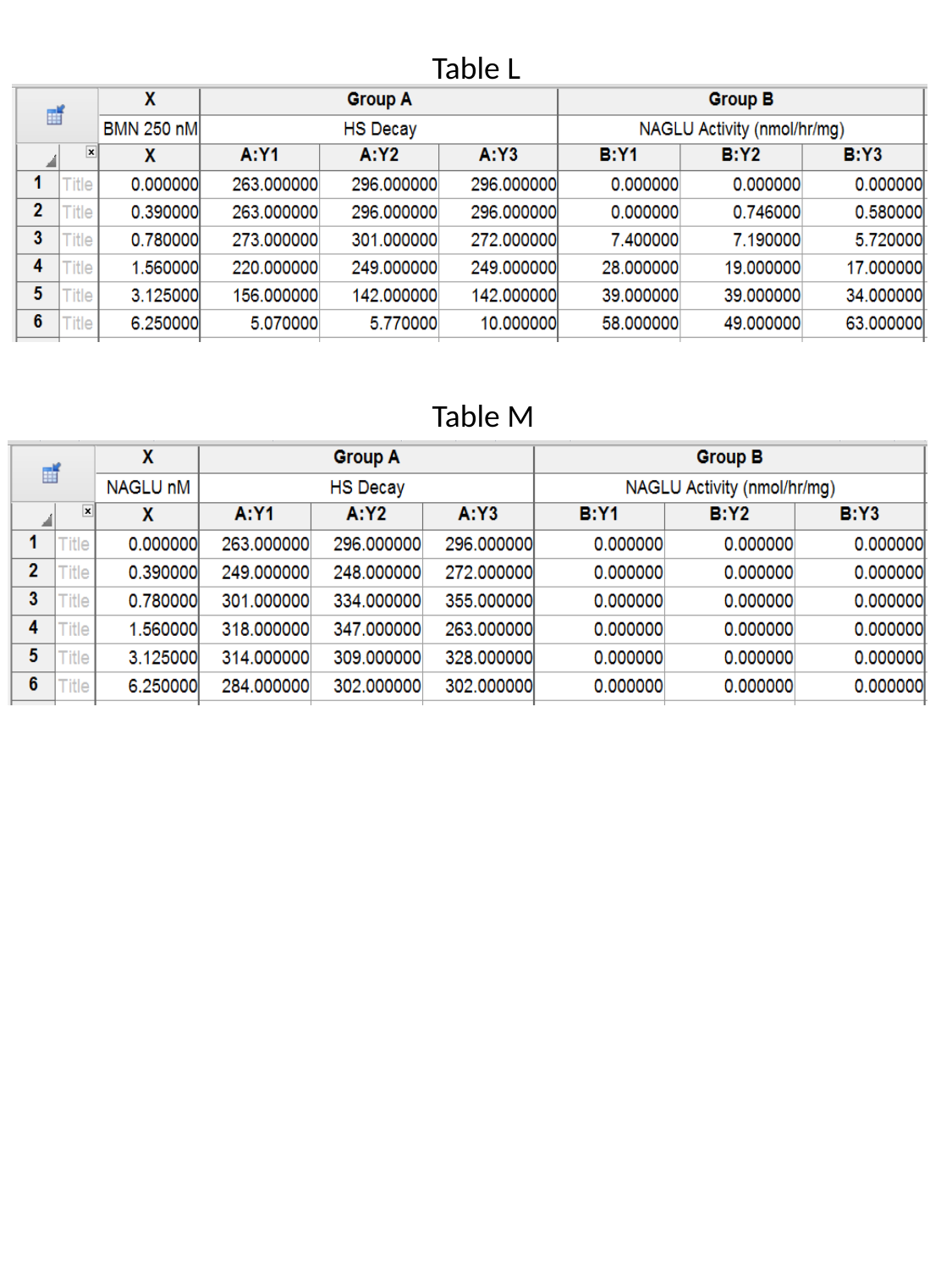

Table L
Table M
